# Modification of Graphene Oxide Membranes by the Incorporation of Nafion Macromolecules and Conductive Scaffolds

**DOI:** 10.3390/nano9040556

**Published:** 2019-04-05

**Authors:** Maria O. Concha-Guzmán, Oscar A. Jaramillo-Quintero, Marina E. Rincón

**Affiliations:** 1 Instituto de Energías Renovables, Universidad Nacional Autónoma de México, Av. Xochicalco S/N, Col. Centro, Temixco 62580, Mexico; 2 Catedrático CONACYT-Instituto de Energías Renovables, Universidad Nacional Autónoma de México, Av. Xochicalco S/N, Col. Centro, Temixco 62580, Mexico; oajaq@ier.unam.mx

**Keywords:** graphene oxide, membranes, Nafion

## Abstract

Stable, reproducible and low-cost graphene oxide (GO)/Nafion (N) membranes were fabricated using electronically conductive carbon paper (CP) matts as a scaffold. The presence of polar groups in the Nafion molecule facilitates the strong interaction with functional groups in the GO, which increases GO dispersion and aids the retention of the composite into the CP scaffold. Distribution of GO/N was carefully characterized by X-ray diffraction work function measurements, Raman and scanning electron microscopy analyses. The performance of these membranes was tested with 1 M NaCl at standard conditions, finding 85% ion removal in the best membranes by a mixed ion rejection/retention mechanism. The Nafion provided mechanical stability and fixed negative charge to the membranes, and its micellar organization, segregation and confinement favored ion rejection in Nafion-rich areas. The good electronic conductivity of these membranes was also demonstrated, allowing for the application of a small potential bias to enhance membrane performance in future studies.

## 1. Introduction

Today, preservation of the environment, the search for renewable energy sources, and the lack of potable water are probably the most significant concerns of society. A promising source of drinking water is the virtually unlimited supply of seawater, but so far, the desalination technology has been too expensive for widespread uses. Nevertheless, as the global water demand continues to increase and water pollution aggravates, desalination, along with wastewater recycling and storm water recovery, could be the latest water supply systems [1,2].

Nanomaterials for water quality management are promising due to their unique physicochemical properties, high removal efficiency, and environmental friendliness [1,3,4]. Photocatalytic nanomaterials [5,6], adsorption nanomaterials [7,8], reducing nanomaterials [9], and new membrane materials in general [10,11,12,13] provide a broad range of possibilities for water treatment, as well as the interfacial materials reviewed recently [14]. In this context, two-dimensional graphene oxide (GO) has been pursued for the preparation of desalination membranes due to its hydrophilicity, molecular sieving properties, and Donnan-type rejection mechanism dominated by electrostatic interactions between fixed membrane charges and mobile ions [15,16,17]. GO permeability to a variety of species with different ionic charge in aqueous and non-aqueous solvents has been reported for membranes obtained by simple vacuum filtration [18,19]. More recently, Na^+^-controlled GO membrane spacings due to non-covalent hydrated cation-π interactions have been shown to exclude other cations that have larger hydrated volumes [20].

GO membranes might have the potential but have not yet been proven to be cost-effective and scalable to a commercial scale in the water treatment industry, since they are mechanically too unstable for real-world membrane operations [16,21]. Delamination significantly increases once graphene becomes oxidized due to the loss of interlayer Van der Walls contributions. In addition, electrostatic interactions and hydrogen bonding between GO and water play significant roles in exfoliating GO in aqueous applications [22]. To solve this problem, the stability of GO membranes has been increased by combining GO with polymeric materials and by functionalizing the membrane support [15,23,24,25,26]. In particular, GO/Nafion composites have been studied in the context of proton exchange membrane (PEM) [27], but no studies were found on desalination purposes, where charge density is important and could modify the membrane performance.

Nafion is formed by a poly(tetrafluoroethylene) main polymer chain (PTFE) of hydrophobic nature, with side chains linked by ether bonds and finished with sulfonic groups (SO_3_^−1^ H^+^) of hydrophilic nature. Molecular dynamics simulations of Nafion films capped by hydrophilic substrates indicate that the number of water molecules, hydronium ions, and sulfonic acid groups accumulates near the hydrophilic substrates. Water clusters present in the hydrated Nafion nanostructure form percolated channels at sufficiently high hydration levels, allowing the transport of ions across the membrane [28], which can either enhance or compromise ion rejection in the desalination purposes.

In this work, we report the preparation of GO/Nafion (GO/N) composites supported by carbon paper. No chemical bonding was pursued, only hydrogen bonding attracting the negative charge of the ionized functional groups of both components. Carbon paper serves as a porous conductive skeleton modified by the deposition of different amounts of GO/N composites. CP-supported GO/N membranes were characterized by various techniques to identify the distribution of GO and Nafion in the inner and outer surfaces of the CP scaffold. Salt retention studies carried out from both sides of the membrane, suggested different rejection mechanisms that depend on Nafion micellar organization, segregation, and confinement. Galvanostatic charge/discharge curves were measured to assess the electronic conductivity of the membranes and their applicability in potential driving applications.

## 2. Materials and Methods 

### 2.1. Materials

Powder graphite (particle size < 20 µm, Sigma-Aldrich, St. Louis, MO, USA) Nafion® (~5% polymer in a mixture of lower aliphatic alcohol and water, Sigma-Aldrich), filter membranes (Millipore HTTP01300 Polycarbonate, 13 mm diameter, pore size 0.4 µm), analytical grade sulfuric acid (H_2_SO_4_, 98%, Fermot, Monterrey, México), sodium nitrate (NaNO_3_, 99.6%, Fermot), potassium permanganate (KMnO_4_, 99%, Fermot), H_2_O_2_ (30% aqueous solution, Fermot), hydrochloric acid (HCl, 37%, Fermot), and carbon paper (Spectracarb 2050L-0850, Shelton, CT, USA, no polytetrafluoroethylene treatment, 18 mΩ cm^2^ electrical resistivity, ~ 40 µm pore size, and 203 µm thickness).

### 2.2. Synthesis of Graphene Oxide

Graphene oxide was prepared from graphite by the Hummers modified method [29]. Briefly, 2 g of graphite, 1 g NaNO_3_, 46 mL H_2_SO_4_, and 6 g of KMnO_4_ were mixed in a flask placed in an ice bath. The flask was warmed to 35 °C and stirred for 2 h. Afterwards, water (92 mL) was slowly added, and 15 min later an additional 77 mL of distilled water and 8 mL of 30% H_2_O_2_ was added. This mixture was filtered through a Buchner funnel and the solid washed with 200 mL of 1 M HCl solution. This solid was transferred to an Erlenmeyer flask for further dilution with distilled water up to 1 L. The resulting solution was centrifuged at 4000 rpm for 10 min, followed by the recovery of the supernatant solution by decantation. This cycle was repeated until a neutral pH was reached. All the decanted suspensions were collected and adjusted to neutral pH to constitute the colloidal solution used in the preparation of the membranes, corresponding to a concentration of 1.2 mg of GO in 25 mL distilled water.

### 2.3. Preparation of CP Supported GO/N Membranes

Inks based on GO colloidal suspension with and without 0.05% Nafion were prepared at room temperature. CP scaffolds were cut into circular shapes, with a diameter of 3.5 cm, washed sequentially in an ultrasonic bath with acetone, distilled water, and alcohol for 15 min in each solution. After drying at room temperature, CP scaffolds were placed on a filter holder on top of a Millipore HTTP01300 filter membrane (Burlington, MA, USA) with a 0.4 µm pore size. A glass funnel was placed and fixed to the filter holder with a large clamp. This system was introduced into a flask, which is connected to a vacuum pump. Once the experimental setup was ready, different volumes of GO/N ink were filtered. A GO/N weight ratio of 1:20 was selected from preliminary testing of ink manageability and composite adhesion to the support.

### 2.4. Electrode Preparation for Galvanostatic Experiments

CP-supported GO/N membranes were cut into 1 cm diameter circles and stuck to Cu foil collectors with double-sided Cu tape. They were immersed in 1 M NaCl electrolyte for 15 min and assembled into symmetric capacitors using a Celgard separator impregnated with 30 µL of 1 M NaCl.

### 2.5. Characterization

Structural characterization was obtained from X-ray diffraction (XRD) studies carried out on a Rigaku DMAX 2200 (Rigaku, Kyoto, Japan) at a range of 0.5–2° grazing angles, with Cu-Kα (λ = 1.5405 Å) radiation. The UV-Vis absorption spectra were measured by Shimadzu UV-3101PC spectrometer (Shimadzu, Kyoto, Japan). Scanning electron microscopy (SEM) images and cross-section energy-dispersive X-ray spectroscopy (EDS) were recorded using a Hitachi US3 (Hitachi, Kyoto, Japan), operated at 5 kV acceleration voltage and a working distance of 9 mm. Raman spectra were collected by a confocal Raman WITec, Alpha 300, 532 nm laser and 100x objective lens (WITec, Ulm, Germany). Work function measurements were performed in a Kelvin probe system (KP Technology SKP5050) under dark ambient condition, using a 2 mm vibrating gold probe with 5.02 eV work function.

To evaluate the desalination performance of the supported GO/N membranes at room temperature and atmospheric pressure, the ionic conductivity was measured by Hanna HI 255 Combined Meter pH/mV and EC/TDS/NaCl instrument (Hanna Instruments, Woonsocket, RI, USA). The homemade gravity filtration system consisted of a modified small centrifugation container to filter 20 mL 1 M NaCl solution through each membrane. The galvanostatic charge/discharge (GCD) test was measured between −0.1 and 0.1 V, using a multichannel electrochemical workstation VMP-3 (BioLogic, Seyssinet-Pariset, France).

## 3. Results and Discussion

### 3.1. CP-Supported GO/N Membranes

**Figure 1:**
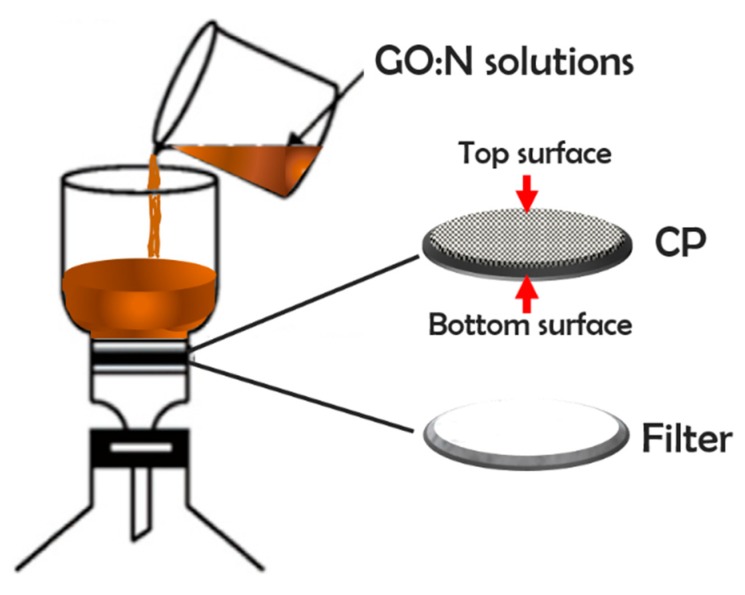
Schematic configuration of the filtration system used to obtain the CP-supported GO/N membranes. Top/bottom surfaces are also shown.

Table 1 indicates the amounts of GO/N colloidal solutions used for vacuum filtration as depicted in Figure 1. The total retention of GO/N composite in the CP support was verified by UV-Vis spectroscopy as the filtrates show spectra similar to distilled water (see Figure S1 in Supplementary Materials). For inks with just one component, the amount of solids retained in the support becomes problematic. Without GO, Nafion is not retained completely in the hydrophobic CP support, permeating into the filtered solution as shown in the images of Figure S2, whereas without Nafion, GO has poor adhesion and leaves residues on the experimental setup (Figure S3). The implications of these observations will be addressed later.

### 3.2. Stability of CP-GO/N Membranes

**Figure 2:**
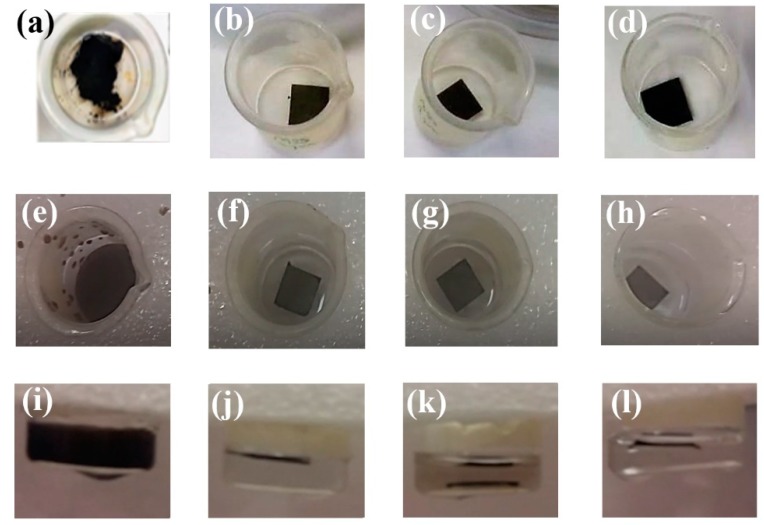
(**a**,**e**,**i**) GO, (**b**,**f**,**j**) 25GO/N, (**c**,**g**,**k**) 50GO/N, and (**d**,**h**,**l**) 75GO/N membranes in distilled water: (**a**–**d**) static mode, (**e**–**h**) top view, and (**i**–**l**) cross view after ultrasonication.

Given that the GO and Nafion materials were simply filtered onto the support substrate, stability was a topic of concern. Figure 2 shows the GO and CP-GO/N membranes under static conditions and after ultrasonication for one minute, in which the exfoliation of GO membranes and the stability of the GO/N coatings on CP scaffolds is clearly visible.

### 3.3. Distribution of GO and Nafion into the CP Support

XRD patterns of GO/N membranes using different grazing incidence angles at the top and bottom surface are shown in Figures S4 and S5. From the XRD data, it is possible to obtain the typical interplanar distance along the c-direction (*d*_002_) and the crystal size and the number of layers estimated from *L*_c_/*d*_002_ of the different membrane components, as listed in Table 2. No considerable changes in GO interplanar distance were observed after Nafion incorporation, and the values obtained are close to those reported in the literature [20]. It was also noticed that the intensity of peaks associated with Nafion and GO increases as the amount of GO/N composite increases, being clearer in the XRD patterns of the bottom surface. These results indicate (i) the impregnation of the CP inner/outer surfaces and (ii) the accumulation of the composite at the bottom surface.

**Figure 3:**
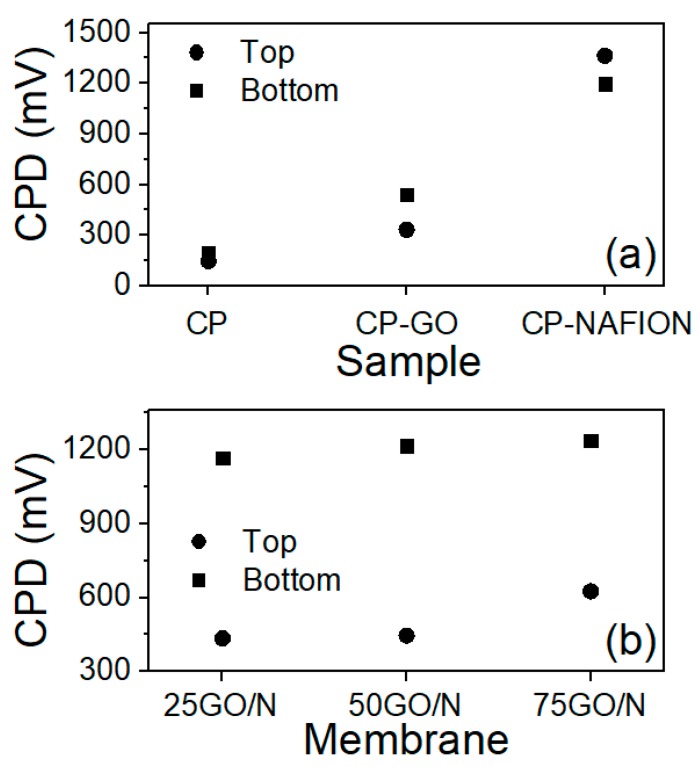
CPD values measured on the top and bottom surfaces of (**a**) CP supported membrane components and (**b**) CP supported GO/N composites.

To further evaluate the distribution of the non-covalent GO/N composite inside the 3D macroporous CP structure, work function (WF) measurements and a Raman analysis were carried out. The GO:N weight ratio was set at 20:1 during ink preparation, and changes along the membrane thickness during vacuum filtration could be due to differences in density. WF is generally estimated indirectly in terms of relative surface contact potential difference (CPD) measurement between two different surfaces following the relation

(1) $$V_{\mathrm{CPD}}=\;\frac{{\mathrm{WF}}_{\mathrm{tip}}-{\mathrm{WF}}_{\mathrm{sample}}}{e},$$

where WF_tip_ is the work function of the probe, and *e* is elementary electron charge. The CPD values of CP, GO, Nafion, and GO/N composites depend on surface composition, type of bonding, and electronic and ionic conductivity. Increasing the oxygen content in GO has been reported to decrease the work function from 5.1 to 4.7 eV [30] due to the abundance of negative charge. Figure 3 shows the CPD values measured at the top and bottom surfaces of the CP-supported components (Figure 3a) and composites (Figure 3b). As Figure 3a indicates, the difference between top/bottom values was relatively small for CP but increased in the CP-supported Nafion (CP-N) and CP-supported GO (CP-GO) membranes. CPD values for CP (−185 mV) become more positive with the impregnation of GO (300–500 mV) and Nafion (1200–1350 mV), indicating a reduction in work function particularly when Nafion macromolecules were present, due to the abundance of negative sulfonic groups. For the GO/N composites, a clear difference between the top/bottom CPD values is obvious in Figure 3b, confirming sensible changes in the GO/N ratio along the membrane thickness. Apparently, Nafion macromolecules accumulated at the bottom surface more rapidly than GO, which was confirmed by SEM images shown in Figure S6. In general, the texture of the top and bottom surfaces was different for all composites.

**Figure 4:**
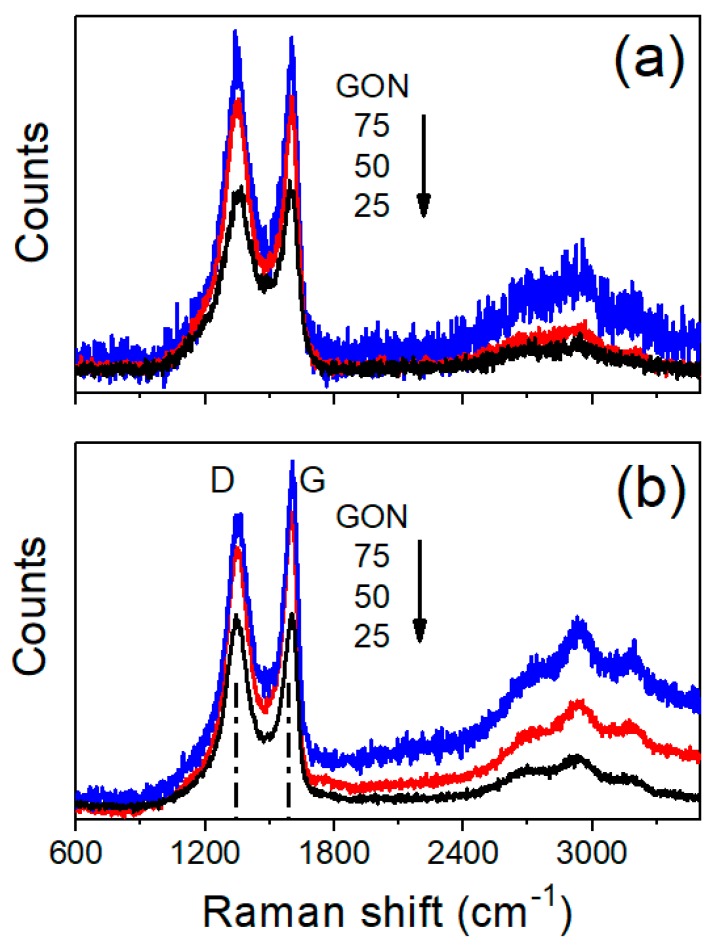
Raman spectra of (**a**) top and (**b**) bottom surfaces of different GO/N composites.

Although the non-uniform distribution of GO and Nafion inside the CP might be a consequence of geometry, steric hindrance, density differences, and surface functionality, the question was whether or not it would impact the mechanism for desalination. According to theoretical simulations, hydrophilic substrates such as GO attract Nafion hydronium ions and sulfonic acid groups enhancing the in-plane water diffusion considerably [28]. On the other hand, the strong π-π interaction between graphitized surfaces (i.e., CP and GO) and the hydrophobic nature of CP, might weaken the GO−Nafion interaction, explaining Nafion enrichment at the bottom surface. Figure 4 shows the Raman spectra of the top and bottom surfaces of the CP-GO/N membranes. Table 3 summarizes the intensities and ratios of the two characteristic peaks exhibited at 1348 and 1596 cm^−1^ corresponding to the D (*I*_D_) and G (*I*_G_) bands, respectively. The analysis of the top surface shows an increase in the *I*_D_:*I*_G_ ratio with the presence of Nafion, which was most likely due to a better dispersion of the GO agglomerates. A large amount of composite favors Nafion reorganization and segregation, allowing the clustering of GO and appearing as a decrease in the *I*_D_:*I*_G_ ratio. This is in agreement with the analysis of the bottom surface, where clear segregation of Nafion macromolecules was documented by CPD values and lower I_D_:I_G_ ratios have been obtained. Raman spectra for GO, CP, and Nafion can be found in Figure S7.

### 3.4. Salt Removal Experiments

**Figure 5:**
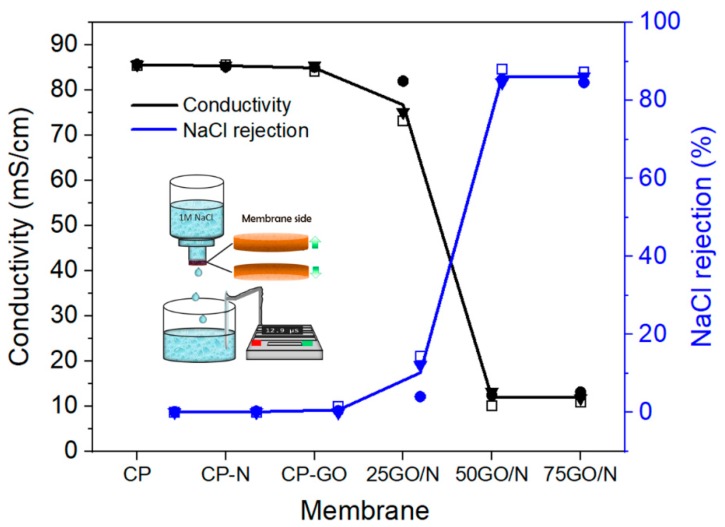
Salt removal in CP, CP-N, CP-GO, and CP-GO/N membranes, bottom side. CP-N and CP-GO showed problems (described further in the text); Nafion was poorly retained in CP supports in the absence of GO, and GO was mechanically unstable in water solutions in the absence of Nafion. Solid lines correspond to the mean value of three samples indicated by the symbols. The inset shows the experimental setup for desalination under atmospheric conditions. The water flux of the 25GO/N and 75GO/N membranes at atmospheric conditions was 0.157 and 0.110 mL cm^−2^ s^−1^, respectively.

**Figure 6:**
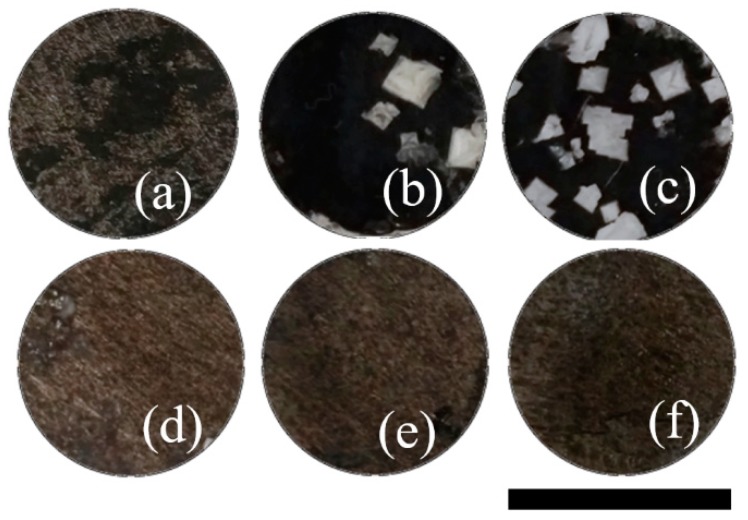
Images of CP-supported membranes after desalination using bottom surface (**a**) CP-25GO/N, (**b**) CP-50GO/N, (**c**) CP-75GO/N, and top surface (**d**) CP-25GO/N, (**e**) CP-50GO/N, (**f**) CP-75GO/N. The scale bar is the diameter of the membrane, 0.9 cm.

The ion removal was evaluated by passing 20 mL 1 M NaCl solution through CP-GO/N membranes with 9 mm in diameter using a homemade gravity filtration system, as detailed in the experimental section and depicted in the inset of Figure 4. The conductivity of the permeate solution was measured after each test, expressing the total ion retention as

(2) $$R_{i}=\frac{S_{if}-S_{ip}}{S_{if}}\times 100\%,$$

where *S_if_* and *S_ip_* are the conductivities in the original feed and permeate solution, respectively. Figure 5 shows the ion removal as a function of GO/N composite, calculated from the ion conductivity differences of the feed and permeate in desalination experiments with the bottom side of the membranes facing the 1 M NaCl. It is evident that ion retention increased with the load of GO/N and reached a plateau for 50GO/N and 75GO/N. Three filtration tests were performed for each membrane, finding less than 2% difference in all of them, except for CP-25GO/N where the deviation was 5%. Similar results were found when the top surface was facing the saline solution. Interestingly, optical images of top and bottom membranes after desalination are quite different, as can be observed in Figure 6. When the bottom side faces the feed, the presence of salt is noticeable and increases as the amount of GO/N increases (Figure 6a–c). On the contrary, crystals are not evident when the top side faces the feed (Figure 6d–f), even though permeate conductivity is similar.

**Figure 7:**
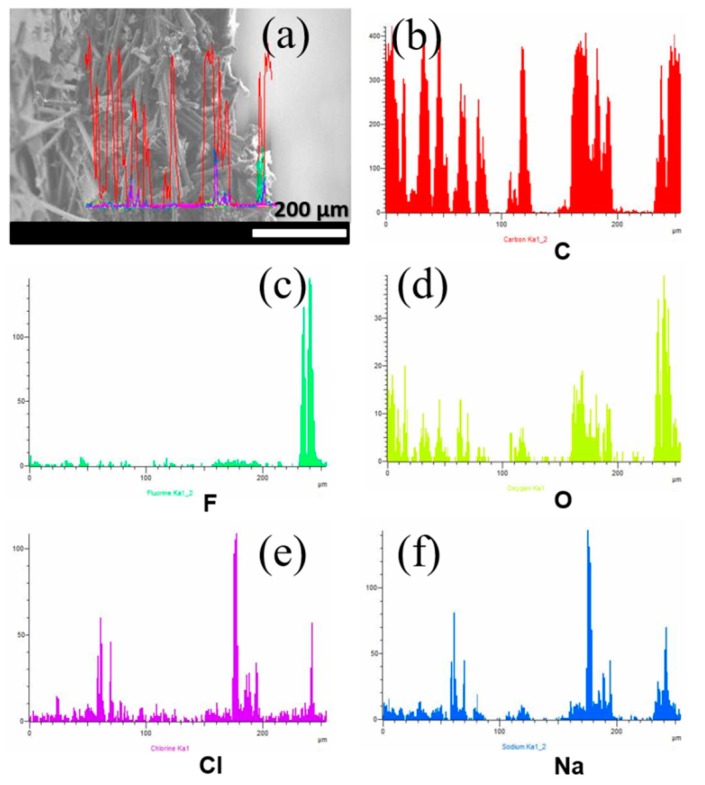
Cross-section EDS line-scan of 50GO/N membranes after desalination experiments with the top surface facing the filtrate: (**a**) SEM image showing the line distributions along the cross section, (**b**) carbon, (**c**) fluorine, (**d**) oxygen, (**e**) chlorine, and (**f**) sodium.

**Figure 8:**
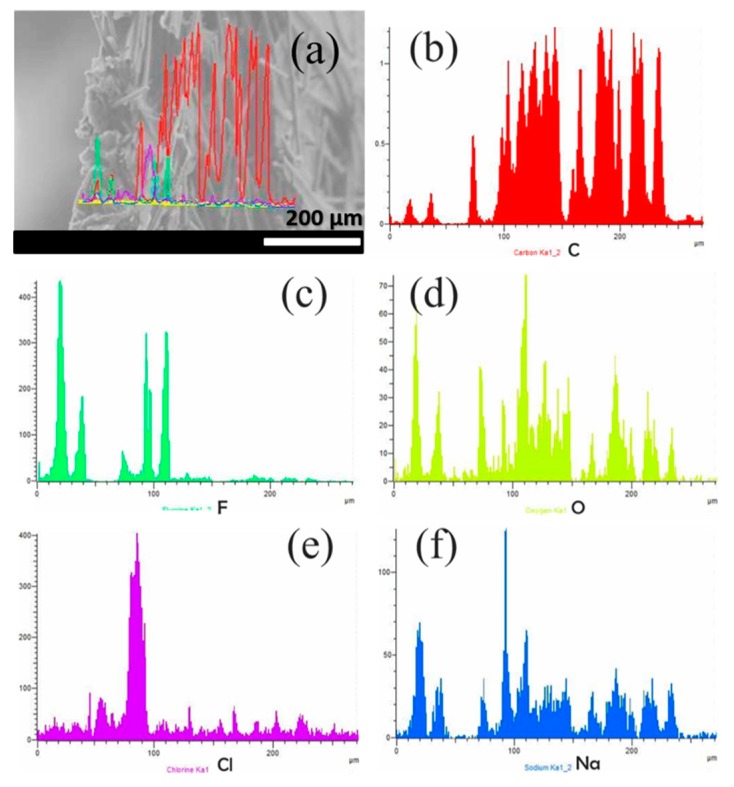
Cross-section EDS of 50GO/N membranes after desalination experiments with the bottom surface facing the filtrate: (**a**) SEM image showing the line distributions along the cross section, (**b**) carbon, (**c**) fluorine, (**d**) oxygen, (**e**) chlorine, and (**f**) sodium.

To identify the places where the salt was retained, a cross-section EDS line analysis of the CP-50GO/N membrane was performed, and the results are shown in Figure 7 and Figure 8. Figure 7 exhibits the element distribution in experiments with the top surface facing the feed. Regarding the membranes’ constituents, carbon and oxygen appeared uniformly distributed along the membrane thickness (Figure 7b,d), in contrast to fluorine (Figure 7c), which appeared in minor amounts along the bulk of the membrane and concentrated mainly at the bottom surface. This result is in agreement with the above-mentioned characterization. The non-homogeneous distribution of Nafion macromolecules allows correlating most of the oxygen in the membrane bulk to GO functionalities. Sodium and chlorine elements (see Figure 7e,f) were strongly correlated along the membrane thickness and closely followed the distribution of O, but not F, suggesting a strong role of GO. On the contrary, when the Nafion-enriched bottom surface was facing the feed (Figure 8), sodium seemed to follow F, O, and C distributions (Figure 8e), whereas chlorine was rejected at the filtrate surface (see Figure 8a) and in regions where the other elements showed a minimum. It is worth emphasizing that crystals outside the membrane were not included in the element distribution.

### 3.5. Charged Membranes

Physical sieving has been discarded as the main removal mechanism in many GO-based membranes, since the hydrated diameters of Cl^−^ and Na^+^ are smaller than the space between adjacent GO nanosheets [15]. Nevertheless, recent work shows that cations can efficiently and selectively exclude other cations that have larger hydrated volumes, but this requires membrane conditioning [20]. The CP-GO/N membranes reported here did not only have interplanar distances above the size of the hydrated ions (*d*_002_ = 0.81 nm > 2diameter_Na^+^_ = 0.72 nm > 2diameter_Cl^−^_ = 0.66 nm), but also wide pores in the CP support. The presence of a polymeric molecule, such as Nafion, did not change the spacings in an obvious way, but the fact that it segregated at one side of the membrane promoted clear differences in salt distribution that could be related to the increase in the amount of fixed negative charge. It seems reasonable to suggest that on the Nafion-enriched side, salt removal was a combination of salt rejection and retention, whereas for desalination experiments facing the top side, salt removal was mainly retention in the bulk of the membrane.

**Figure 9:**
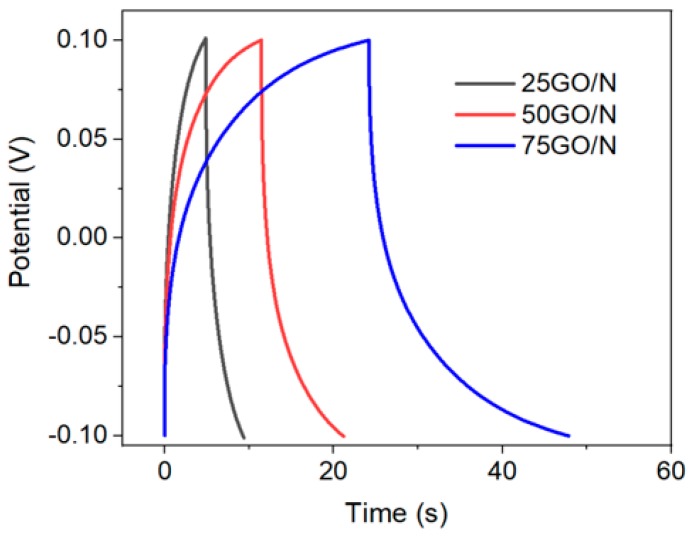
Galvanostatic charge/discharge curves of CP-supported GO/N membranes tested in a Swagelok cell using 30 µL 1 M NaCl.

Moreover, by taking advantage of the electronically conductive CP support, galvanostatic charge/discharge curves were obtained in 1 M NaCl electrolyte using a symmetric two-electrode cell (the details of electrode and cell preparation are given in the experimental section). Since just 30 µL 1 M NaCl were used as the electrolyte, the galvanostatic curves at current densities from 5 to 25 A/g were obtained in a very narrow potential window (from −0.1 to 0.1 V) to avoid electrolyte depletion. Figure 9 shows the non-linearity of the charge and discharge curves, where the energy storage during charging was not completely discharged. It is evident that phenomena more complex than double layer contributions took place. It is also apparent that regardless of the porosity and the dielectric nature of GO/N coatings, the membranes had a good electronic conductivity. Under the testing conditions, charge density accounted for 0.4 to 9.62 mM NaCl/g, which is less than 0.014% the membrane capacity (see Figure 9). Under this small bias, the 75GO/N membrane was more efficient than the 50GO/N, and further optimization of the filtration device can enhance salt rejection versus retention inside the membrane and it could be used as a continuous cell. Table 4 summarizes the results obtained.

## 4. Conclusions

We report the preparation of GO/Nafion composites supported by electronically conductive carbon paper. Nafion macromolecules provided mechanical stability and a fixed negative charge to the membranes, and its micellar organization, segregation, and confinement favored ion rejection in Nafion-rich areas. Optimization of the amount of composite (i.e., filler volume) led to 85% salt removal under gravity filtration through a mixed mechanism of rejection and retention, as indicated by linear EDS analysis along the thickness of the membranes. Although Nafion enrichment seems to promote ion exclusion, a small bias would be beneficial and can easily be implemented due to the good electronic conductivity of the CP-supported membranes, as demonstrated by galvanostatic charge/discharge experiments.

## Figures and Tables

**Table 1:** Component ratios for preparation of membranes with 3.5 cm diameter.

Membrane	Filtration volumes	GO (mg)	Nafion (mg)
25GO/N	25 mL GO + 12.5 mL Nafion (0.05%)	1.2	0.06
50GO/N	50 mL GO + 25 mL Nafion (0.05%)	2.4	0.12
75GO/N	75 mL GO + 37.5 mL Nafion (0.05%)	3.6	0.18

**Table 2:** Values of the diffraction peak position, interplanar distance, crystal size, and estimated number of layers for GO, CP, and Nafion.

Sample	Diffraction Peak 2θ (°)	Interplanar Distance *d*_002_ (nm)	Size Crystal *L*_c_ (nm)	Number of Layers *L*_c_/*d*_002_
GO ^1^	11	0.79 ± 0.1	-	-
GO/N	11.1	0.81	9.7	~12
CP	26.3	0.33	9.8	~30
Nafion	17	0.51	7.7	~15

^1^ Taken from [20].

**Table 3:** Raman intensities of D, G, and D:G ratios measured at top and bottom surfaces of membranes.

Membrane	Top			Bottom		
	*I*_D_	*I*_G_	*I*_D_/*I*_G_	*I*_D_	*I*_G_	*I*_D_:*I*_G_
CP	1.08	1.11	0.93	-	-	-
GO	1.38	1.46	0.95	-	-	-
25GO/N	0.18	0.18	1.00	0.69	0.69	1.00
50GO/N	0.25	0.25	1.00	0.89	1.01	0.88
75GO/N	0.29	0.30	0.96	0.99	1.15	0.86

**Table 4:** Capacitance, *C*; charge density, *Q*; and salt removal, *C*_NaCl_, of CP-supported GO/N membranes obtained from the integration of the galvanostatic charge curve, *Area*_GCC_, under a small potential bias of 200 mV.

Sample	*m*_T_ (mg)	*J* (A g^−1^)	*Area*_GCC_ (V s)	*C*^1^ (F/g)	*Q*^2^ (C/g)	*C*_NaCl_^3^ (mM/g)
25GO/N	0.2	5	0.779	0.195	39	0.4
50GO/N	0.4	25	1.912	2.39	478	4.95
75GO/N	0.6	25	3.977	4.64	928	9.62

^1^ 1*C* = 2*J*Area_GCC_/(ΔU)^2^; ^2^ *Q* = C*ΔU; ^3^ *C*_NaCl_ = *Q*/96,485.

## Supplementary Materials

The following are available online at https://www.mdpi.com/2079-4991/9/4/556/s1. Figure S1: UV-Vis absorption spectra of the different GO/N colloidal solutions before (BF) and after (AF) gravity filtration. GO, Nafion 5% and distilled water are included for comparison; Figure S2: Photographs of Nafion permeation after vacuum filtration through CP support; Figure S3: Photograph of GO remains after removing the CP/GO membrane from the gravity filtration set up; Figure S4: XRD patterns of CP supported 25GO/N, 50GO/N, and 75GO/N membranes using a grazing angle of 0.5°. (a) Top and (b) bottom surface as explained in Figure 1. The dominant graphitic plane (102) at 2θ = 25.1° is mainly from the CP support, whereas GO and N show poorly resolved and low intensity peaks at 2θ = 11.1° and 17°, respectively; Figure S5: XRD patterns of CP supported 75GO/N membranes taken at different grazing angles. Analysis taken at the top (a) and bottom (b) membrane surface. Here again, it is evident that GO and N peaks are more intense in the bottom half than in the top half of the membranes. In both figures the intensity of GO and N peaks increases as the grazing angle increases, confirming impregnation of the inner surfaces of the CP support; Figure S6: SEM images of the membranes (a–c) top and (d–f) bottom surfaces: (a,d) 25GO/N; (b,e) 50GO/N; (c,f) 75GO/N, Figure S7: Raman spectra of GO, CP and Nafion.
