# Supplementary material for: Modification of Graphene Oxide Membranes by the Incorporation of Nafion Macromolecules and Conductive Scaffolds
Source: Nanomaterials (Basel). 2019 Apr 5;9(4):556. doi: 10.3390/nano9040556 (PMC6523627; doi:10.3390/nano9040556)
Supplement: Supplementary file 1 [file nanomaterials-09-00556-s001.pdf]

Supplementary Materials

Modification of Graphene Oxide Membranes by the Incorporation of Nafion Macromolecules and Conductive Scaffolds

Maria O. Concha-Guzmán [1,*], Oscar A. Jaramillo-Quintero [2] and Marina E. Rincón [1,*]

[1] Instituto de Energías Renovables, Universidad Nacional Autónoma de México, Av. Xochicalco S/N, Col. Centro, 62580 Temixco, Mor., México

[2] Catedrático CONACYT-Instituto de Energías Renovables, Universidad Nacional Autónoma de México, Av. Xochicalco S/N, Col. Centro, 62580 Temixco, Mor., México; oajaq@ier.unam.mx

* Correspondence: maocg@ier.unam.mx (M.O.C.-G.); merg@ier.unam.mx (M.E.R.); Tel.: +52-777-362-0090 (M.E.R.)

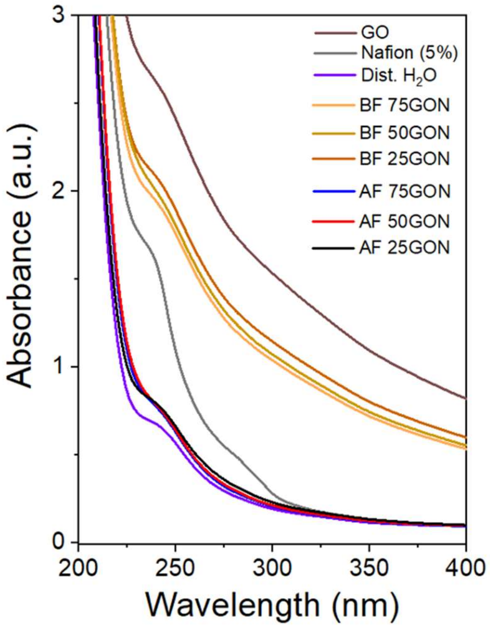

Figure S1. UV-Vis absorption spectra of the different GO/N colloidal solutions before (BF) and after (AF) gravity filtration. GO, Nafion 5% and distilled water are included for comparison.

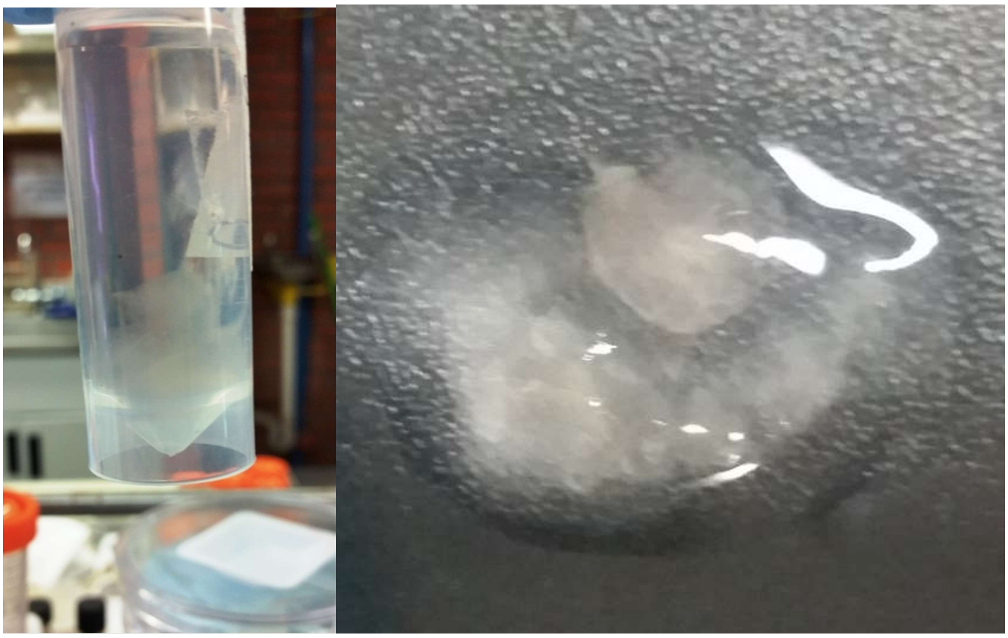

Figure S2. Photographs of Nafion permeation after vacuum filtration through CP support.

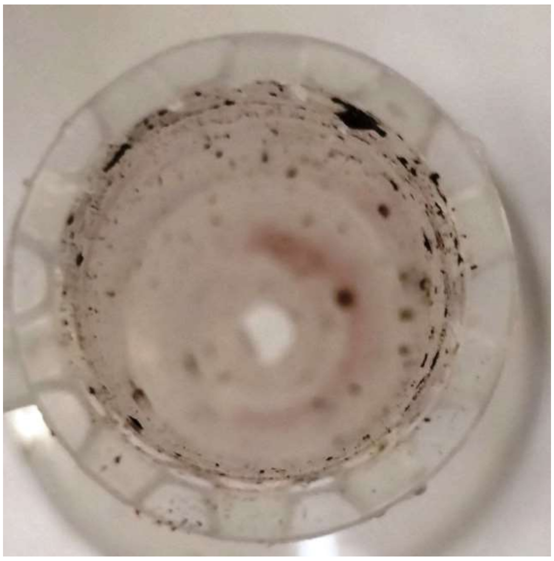

Figure S3. Photograph of GO remains after removing the CP/GO membrane from the gravity filtration set up.

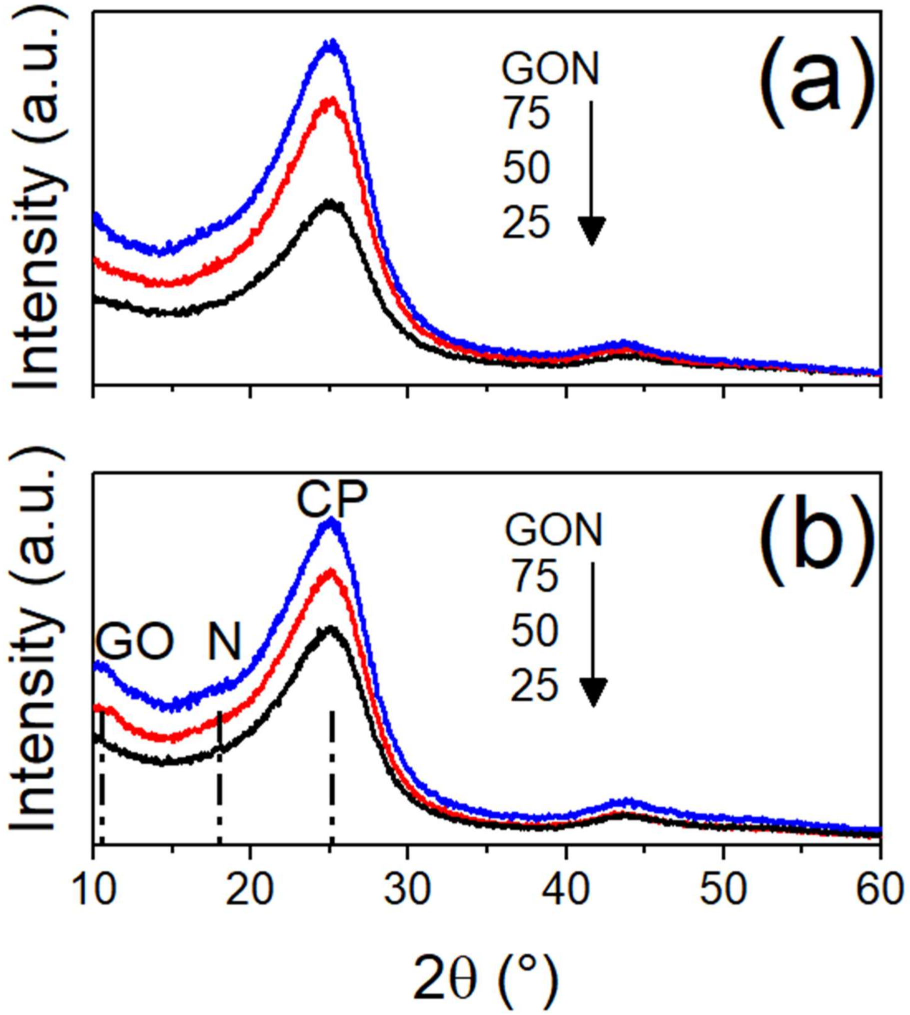

Figure S4. XRD patterns of CP supported 25GO/N, 50GO/N, and 75GO/N membranes using a grazing angle of 0.5°. (a) Top and (b) bottom surface as explained in Figure 1. The dominant graphitic plane (102) at 2θ = 25.1° is mainly from the CP support, whereas GO and N show poorly resolved and low intensity peaks at 2θ = 11.1° and 17 °, respectively.

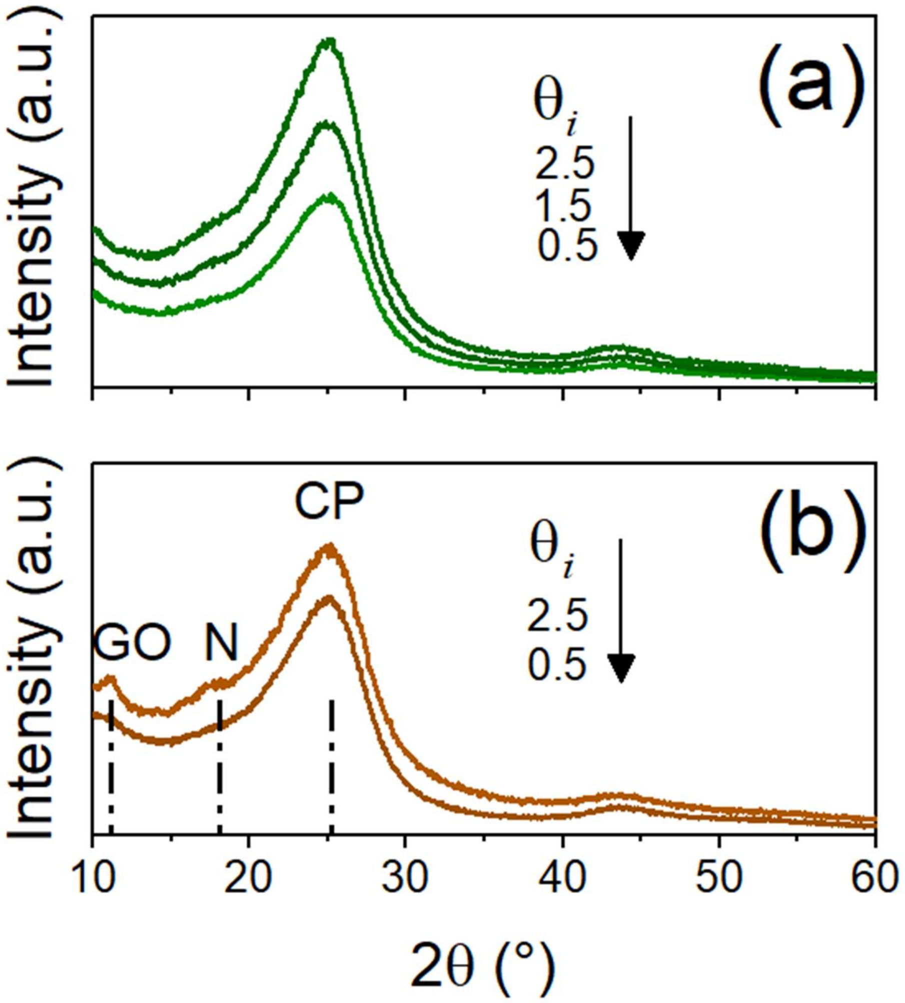

Figure S5. XRD patterns of CP supported 75GO/N membranes taken at different grazing angles. Analysis taken at the top (a) and bottom (b) membrane surface. Here again, it is evident that GO and N peaks are more intense in the bottom half than in the top half of the membranes. In both figures the intensity of GO and N peaks increases as the grazing angle increases, confirming impregnation of the inner surfaces of the CP support.

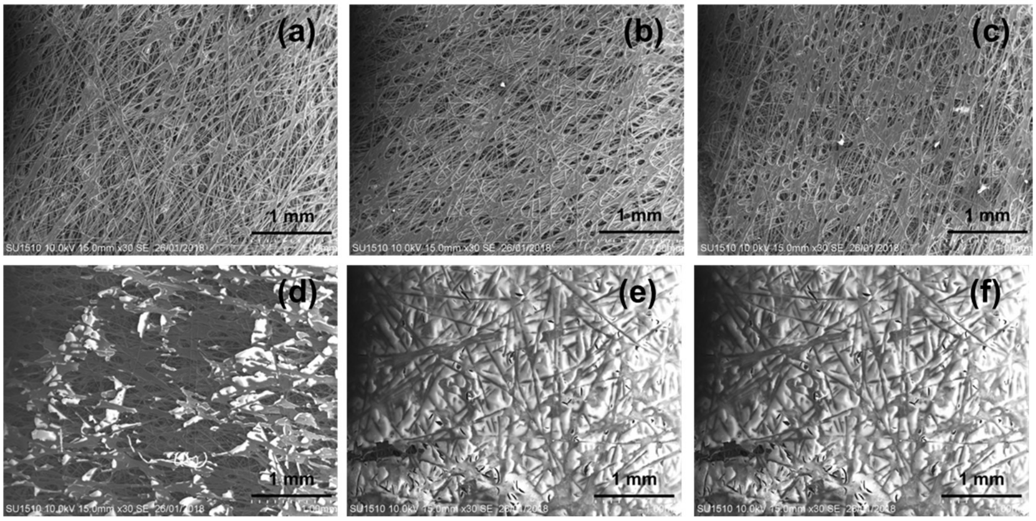

Figure S6. SEM images of the membranes (a-c) top and (d-f) bottom surfaces: (a, d) 25GO/N; (b, e) 50GO/N; (c, f) 75GO/N.

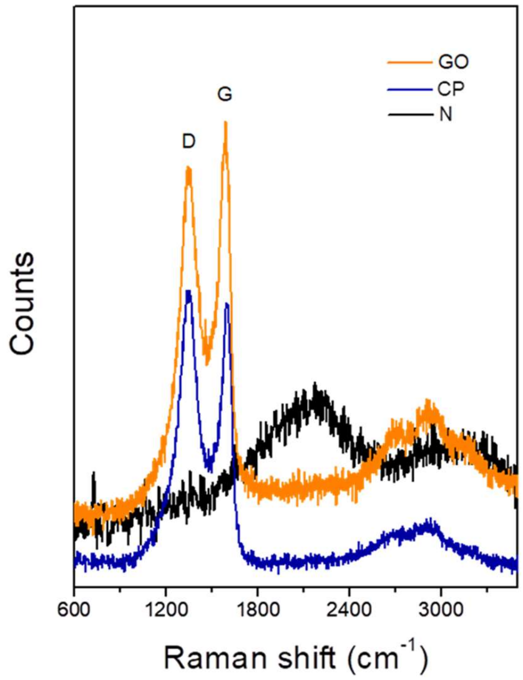

Figure S7. Raman spectra of GO, CP and Nafion.
